# Treatment of oral hyperpigmentation and gummy smile using lasers and role of plasma as a novel treatment technique in dentistry: An introductory review

**DOI:** 10.18632/oncotarget.14887

**Published:** 2017-01-29

**Authors:** Nayansi Jha, Jae Jun Ryu, Rizwan Wahab, Abdulaziz A. Al-Khedhairy, Eun Ha Choi, Nagendra Kumar Kaushik

**Affiliations:** ^1^ Department of Oral and Maxillofacial Implantology, Graduate School of Clinical Dentistry, Korea University, Seoul, South Korea; ^2^ Department of Zoology, College of Science, King Saud University, Riyadh, Saudi Arabia; ^3^ Plasma Bioscience Research Centre, Kwangwoon University, Seoul, South Korea

**Keywords:** excessive gingival display, gingival hyperpigmentation, laser surgery, melanosomes, non-thermal plasma

## Abstract

Gingival hyperpigmentation and the condition known as gummy smile are very common dental cosmetic problems. Gingival hyperpigmentation arises due to the excess presence of melanin in certain regions of the gums. In the case of gummy smile, more than the required amount of gingival tissue is exposed upon smiling. An aesthetically pleasing smile should expose only a negligible amount of gingival tissue. Gummy smile and gingival hyperpigmentation can have detrimental effects on the aesthetic quality of a smile, and thereby a wide variety of treatment options must be taken into consideration depending patient outcome objectives. The use of a laser as a treatment modality is considered to be a promising option for such cases. We aim to explain the effects of using a laser on the gingiva and discuss the advantages and disadvantages of this type of treatment and the resulting alteration of the genetic composition of the gingival tissue. This article reviews the histological aspects and biological effects of a laser treatment for oral hyperpigmentation and gummy smile and analyzes the use of the laser as a modality to improve the smiles of people with hyperpigmentation and excessive gingival display. We also attempt to provide insight into the use of plasma as a novel technology for medical and dental research and its future implications with regard to, dental soft tissue procedures.

## INTRODUCTION

Gingival hyperpigmentation is a benign condition which occurs due to excessive melanin deposition (ethnic/physiologic) in the basal and supra-basal epithelial layers. Clinical pigmentation may not be a medical problem, but it can be of cosmetic concern to patients.

Another common cosmetic problem is gummy smile, referring to an excessive amount of gingival display. The appearance of a gummy smile and/or hyperpigmentation of the gingiva can affect the confidence of patients and result in psychological stress.

Various treatment modalities are currently available, such as surgery as well as electrocautery and orthodontic treatments. However, treatment with laser has emerged as the quickest and most comfortable option. Despite the high cost and sophisticated equipment requirements, laser ablation has become an essential dental cosmetic treatment [1–52].

With regard to dentistry, laser energy has been analyzed and several studies have shown that dental lasers have some degree of affinity for different tissue components. Certain laser configurations specially target soft tissues with affinity toward hemoglobin and melanin, making lasers highly efficient when used to treat soft tissue problems [3]. In this article, we discuss the treatment of gingival hyperpigmentation and gummy smile using lasers and their effects at the molecular level. We also analyze the benefits of non-thermal plasma as a therapeutic modality [53–69]and its potential implications for minor surgeries, when integrated with laser technology.

### Current state of laser technology in dentistry

At present, advancements in lasers are occurring at a very rapid rate. New lasers with a variety of characteristics are being used in medicine and dentistry. Designs using optical fiber with a diameter of 300μm and semiconductor diode lasers are most commonly used [20, 43]. Lasers in the middle and far-infrared regions are typically used in the health care industry for hard and soft tissue procedures. Two key advantages of laser-based systems are high sensitivity and the lack of the attendant risks of ionizing radiation, thus allowing their successful use in dentistry.

**Figure 1:**
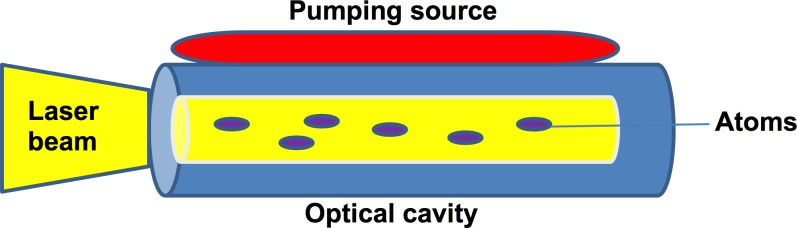
The laser diode with an active medium and a pumping source enclosed in an optical cavity Electrical discharges/light within the diode result in laser activity.

Generally, lasers utilize an optical cavity with, an enclosed (active) medium and a pumping source. The active medium is in inactive state initially, reaching an excited state when it is pumped by the pumping source. For efficient laser activity, the lasing medium is pumped by intense flashes of light and electrical discharges, which creates a collection of atoms in an excited state. It can be in the form of a gas or a solid depending on the active lasing medium used (Figure 1). In dentistry, currently popular active mediums are argon and CO_2_ in the case of gas media, with a garnet crystal of yttrium and aluminum, known as YAG lasers, used in the case of a solid medium [23].

### Lasers for soft tissue procedures

Various soft tissue procedures can be performed with lasers. The main reasons for their use are to reduce bleeding intra-operatively and to minimize the pain post-operatively compared to conventional techniques such as electrosurgery. For minor soft tissue surgery, a wide variety of lasers can be used, ranging from the Er: YAG 2940 nm type, which provides the least hemostasis, to the Nd: YAG 1064nm type, which is used to provide the highest level of hemostasis [43].

High-intensity lamps (Xenon) and selective lasers such as those based on gallium (wavelengths: 600-900nm) have found wide use in dentistry. They provide excellent results painlessly in relatively less time and with high efficiency. Lasers can be used as an adjunct to surgical treatment or can be used independently.

## GINGIVAL HYPERPIGMENTATION

The gingiva consists of melanocytes, and the deposition of excessive melanin (non-hemoglobin derived pigment) by melanocytes (in the basal and supra-basal epithelia) can result in hyperpigmentation. Several different conditions, such as Peutz-Jeghers syndrome, Albright syndrome, melasma and Graves’ disease, can result in high secretion levels of oral melanin.

Clinical melanin pigmentation of the gingiva is not a medical problem, although the presence of black gums may cause aesthetic issues, especially if the pigmentation is visible during communication [4].

### Factors influencing gingival melanin activity

**Figure 2:**
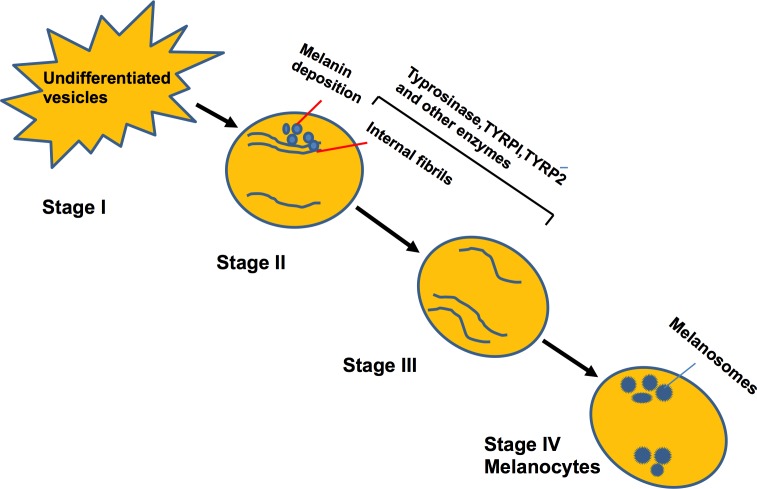
Cycle depicting the maturation of melanocytes and formation of melanin Enzymes are involved in the differentiation of unstructured vesicles to mature and heavily pigmented melanocytes.

Melanocytes can synthesize melanin (melanosomes), using various proteins and enzymes, necessary for the maintenance of melanosome, and for conversion of pre-melanosomes into the mature type. In mammals, there is a chain of events which facilitates the conversion of undifferentiated vesicles (stage I) to dense (stage IV) heavily pigmented melanocytes [38]. Various pigment-cell-specific enzymes are involved in this process, including tyrosinase and tyrosinase-related proteins TYRP1 and 2 (Figure 2).

**Figure 3:**
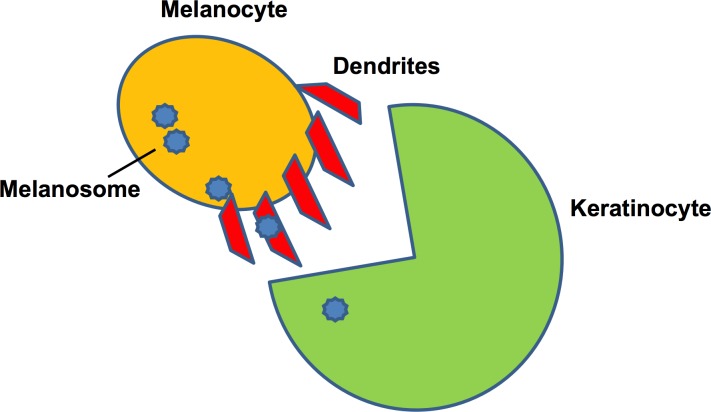
Keratinocyte-melanin unit showing the transfer of melanosome by the dendritic processes of melanocytes The microtubules facilitate of dendritic processes facilitate this transfer.

Inside a ‘keratinocyte-melanin unit’, coordinated events occur whereby mature melanosomes are transported to the keratinocytes using elongated melanocytic dendrites as a transport medium (Figure 3). Within each keratinocyte, the melanin (contained in nuclei) acts as an ultraviolet shield for the DNA [18, 39, 40]. Because the activity of melanosomes is directly interlinked with that of keratinocytes, the occurrence of pigmentation is strongly influenced by keratinocytes.

### Why hyperpigmentation occurs

It has been suggested that although pigmentation under normal conditions is genetically determined, its distribution in the mouth can be due to secondary influences and environmental factors. All individuals, whether lightly or darkly pigmented, have an identical number of melanocytes in any given region of the mucosa, but it has been observed that cells with melanin are present in connective tissue of individuals who have very high amounts of melanin pigment. These cells are macrophages that have engulfed the melanin pigment [13, 41].

### Laser treatment for gingival hyperpigmentation

The treatment of gingival hyperpigmentation using a laser is gaining popularity. As noted earlier, most lasers used in dentistry, are of the infrared type. Research has shown that in cases of chronic periodontitis, the easy removal of infected epithelium can be achieved using a pulsed diode laser (810 λ). Using a laser, the gingival shape can be modified, the dependence on local anaesthesia is decreased, and the lowest amounts of post-operative pain and inflammation are experienced by the patient [1]. Shenawy et al. [1] tested a 320μm, 980nm wavelength diode laser for the depigmentation of gingiva and reported easy (80%) depigmentation with minimal tissue penetration and slight bleeding. A white fibrin slough was seen after 24 hours in the patients due to the formation of a thick coagulation layer (biological wound dressing) on the treated surface produced by the “hot tip” of the diode laser fiber optic. This is characterized as a laser wound, which eventually heals [1, 7].

The production of excess melanin may be a gingival trait, and it is found mostly in certain ethnic groups [11]. It is not known whether melanin hyperpigmentation is a defense mechanism or not. Hossam et al. studied the role of melanin pigmentation in gingivitis and found that upregulation of the growth factor STK 38 gene in association with gingival melanin can alleviate gingival inflammation [2].

### Method of laser action

For pigmented lesions, the laser treatment must be done in a fixed, target region. Photothermolysis (the conversion of light to heat) should be achieved, at which point the laser action should be directly on the melanocytes (containing melanin).

Diode lasers exhibit thermal effects using the hot-tip effect caused by heat accumulation at the end of the fiber; thus, depigmentation is performed using only the tip of the fiber. Lasers allow for controlled cutting with a limited depth of necrosis. This is due to their inherent ability to be absorbed within the chromophores (wavelength-specific light-absorbing substances) with a specific target tissue. In this way, they cause tissue-specific ablation in a layer-by-layer and cell-by-cell manner [49].

Diode lasers can be used successfully to treat a hyper-pigmented inflamed periodontal sulcus and for the removal of bacteria from sulci regions, as the laser energy is not absorbed fully by the hard tooth structure, which mainly contains hydroxyapatite, but is easily absorbed by diseased soft tissue containing melanin, hemoglobin and other chromophores. The laser parameters (e.g., the pulse duration) must be modified based on many factors, with tissue pigmentation being one major factor [5].

As a result of both heat diffusion and light diffusion through scattering, two main effects appear from irradiation with the previously mentioned parameters. These are a coagulated and a necrotized layer, together with an underlying area that is only partially coagulated, with the extent of irreversibly damaged melanocytes gradually decreasing with the depth in this underlying area. This may be a crucial point with regard to the mechanism of action [16, 30]; although the remaining melanocytes may be fewer than normal, they may produce enough melanin to re-pigment the newly formed mucosa during and after the healing period, thus equalizing the pigmentation with that of the surrounding tissue [9].

### The different types of lasers used on gingiva

Different types of lasers, such as the carbon dioxide (CO_2_) laser, Nd: YAG laser [4] semiconductor diode laser, argon laser, Er: YAG laser and the Er,Cr: YSGG laser have been used with excellent results for gum depigmentation. Nd: YAG and diode lasers can selectively remove gingival pigmentation due to their high absorption rate in melanin. However, the CO_2_ laser can at times coagulate the site of surgery and cause postoperative complications. The use of an erbium laser is beneficial as it has a low penetration depth and fewer side effects than those produced by other lasers, with less damage to bone as well [14].

Gallium (Ga), arsenide (Ar), aluminum (Al), indium (In) and other elements can be used to convert electrical energy into light energy. For this reason, these elements have been used for the construction of semiconductor diode lasers [3]. Diode laser are versatile lasers which can be used at the three wavelengths of 980 nm, 810 nm and, more recently, 940 nm [17] as a means of soft tissue treatment.

Soliman et al. [10] reported that the soft tissue diode laser wavelength results in maximal tissue absorption and minimal penetration as compared to minimal tissue absorption and maximal penetration when using an Nd:YAG Laser. Hence, the use of diode laser is a much safer mode of treatment.

In a comparison study [12] between treatment modalities using a scalpel, electrosurgery and a laser, it was observed that the use of a laser resulted in less re-pigmentation as compared to the scalpel technique, as the penetration and necrosis of melanin cells by the laser was closer to the desired depth, thereby reducing instances of recurrence.

Gupta reported the use of a semiconductor diode laser (emitted in gated-pulsed mode) for the treatment of hyperpigmentation. Patients were treated without anaesthesia and laser ablation was done from the mucogingival junction towards the free gingival margin, including the papillae in overlapping circles. Much attention was devoted to avoid passing the laser beam over tooth structures and mucosa. With this technique, successful treatment was achieved for hyperpigmentation removal [3].

The Er:YAG laser has high absorption through water; hence, using this type of laser results in less tissue degeneration and very thin surface interaction. Owing to this characteristic, the use of an Er: YAG laser can minimize damage to deep tissues and prevent scarring from the application of the laser. Lee et al. [8] also found it to favorable compared to other types of lasers.

Er: YAG laser irradiation is known to stimulate the proliferation and secretion of gingival fibroblasts, suggesting that this type may offer therapeutic benefits for tissue repair [36]. Rosa et al. [21] carried out a treatment with an Er: YAG laser, known for its smooth application and high bactericidal effect, and concluded that it is one of the most promising lasers. Tal et al. [11] investigated the use of the Er: YAG laser and reported the speedy recovery of patients due to the narrow zone of thermal disruption. There were no reported cases of re-pigmentation.

In another study, Ozbayrak et al. [7] reported that treatment with a CO_2_ laser causes minimal damage to the periosteum and gingival bone and that it offers a means by which to remove a very thin epithelial layer. The laser wound is a sterile inflammatory reaction that heals more slowly than a surgical wound but with less postoperative pain. A one-step laser treatment is effective for the elimination of any dark-colored pigmented area in the soft tissue, with re-epithelialization occurring in less than a month [7]. Esen et al. [23] reported the use of a CO_2_ laser for the depigmentation of gingiva in pulsated mode with a successful outcome.

Monteiro et al. treated a case of smokers’ melanosis using a CO_2_ laser in one session using pulsated mode [15]. Several researchers reported the use of lasers in a pulsed mode, while others reported the use of the continuous mode. When the laser is used on soft tissues, cells are ruptured, which results in the release of debris known as char (carbonized tissue due to laser burn). The formation of char is greater when the laser is used in continuous mode than it is in the gated/pulsated mode. If char accumulates, it can result in a rapid temperature jump to 1500-2000 degrees, which can cause extensive thermal damage and easily eliminate the gingiva [42].

Chandna et al. [6] compared and evaluated pain using electrosurgery and a diode laser for a depigmentation treatment and found that electrosurgery can result in uncontrolled necrosis of the tissue whereas lasers offer a controlled range of treatment, making them a better choice than electrosurgery. Elavarasu et al. [19] also reported similar results in a comparison between electrocautery and laser therapy.

Chawla et al. used [49] LLLT (low-level laser therapy) as a wound healing measure after depigmentation and achieved excellent results up to the third day, after which no significant differences were found. This indicates that a low-level laser is beneficial, but the effects were comparable with, but not significantly different from, those associated with other modes of laser treatment.

The outcomes of laser treatments differ from patient to patient and are dependent on the various parameters used, such as the dose, time and method of radiation.

### Biostimulatory effects of a laser treatment

**Figure 4:**
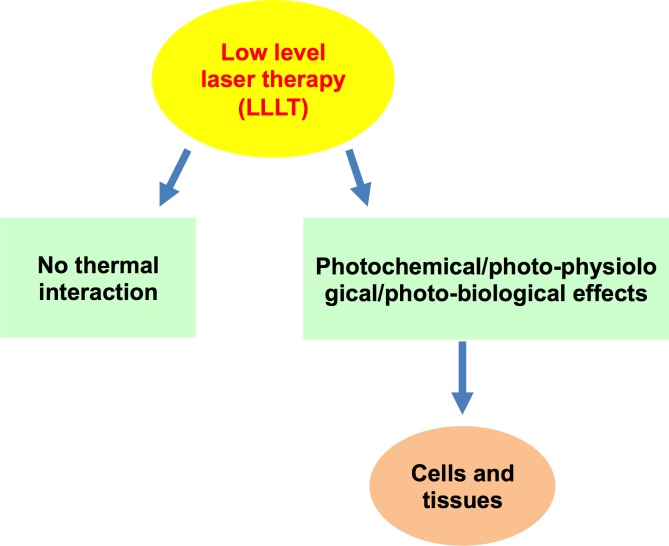
Low-level laser therapy (LLLT) has bio-stimulatory effects on the cells and tissues

**Figure 5:**
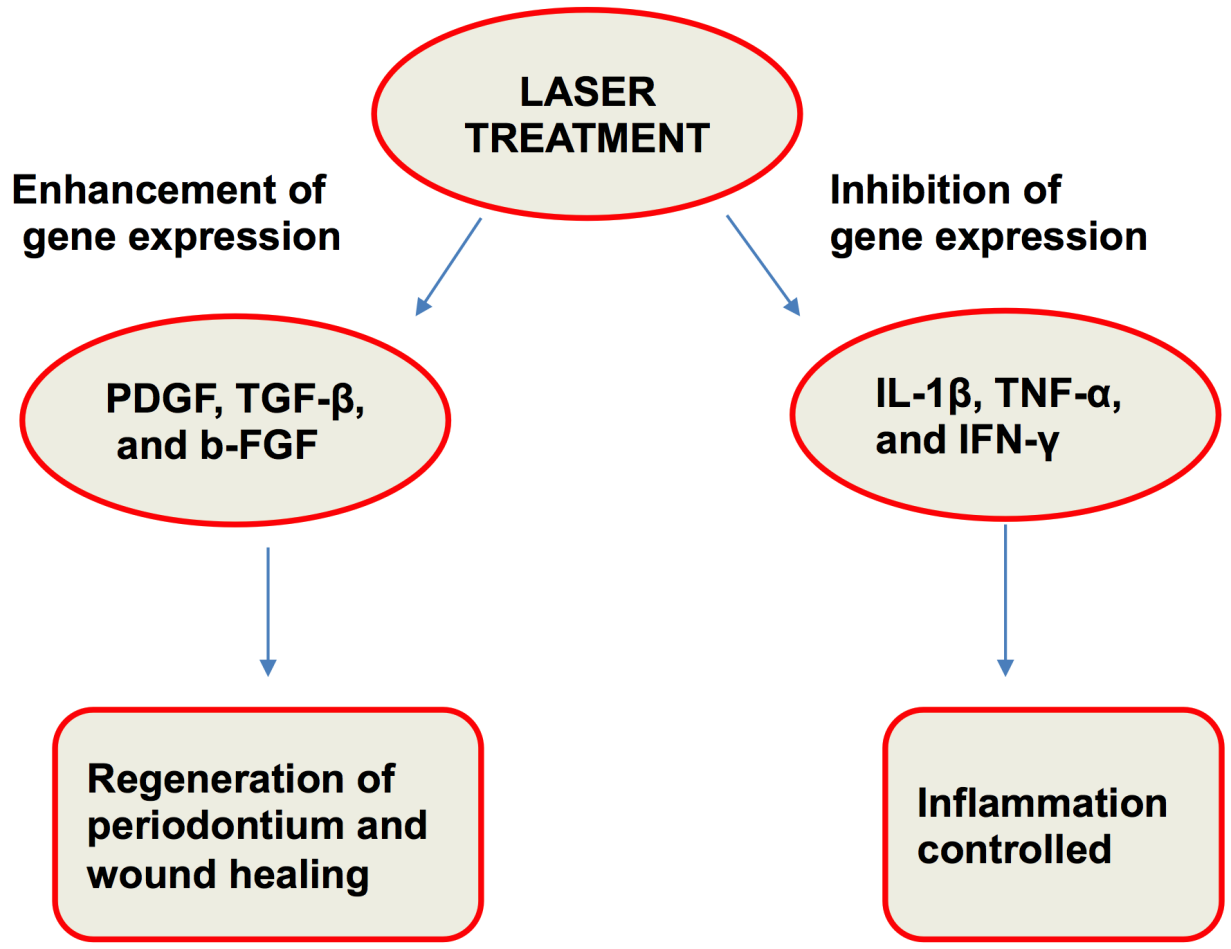
Laser treatment results in regeneration and wound healing with inflammation control

Amorim et al. [48] studied gingival healing after a laser treatment (Figure 4). Laser therapy plays an important role in wound healing and repair, but the exact biological mechanism has not been studied. However, many researchers have attempted to elucidate the biological mechanism, such as Safavi et al. [37], who studied gene expression characteristics, after a laser treatment involving rat gingiva. A low-level He-Ne laser was used, and laser irradiation significantly inhibited the gene expressions of IL-1β and IFN-γ and significantly increased the gene expressions of PDGF and TGF-β (Figure 5).

LLLT can serve as a type of anti-inflammatory therapy with effects comparable to those of nonsteroidal anti-inflammatory drugs (NSAIDs). It was found that the concentrations of prostaglandin ES2 (PGE-2), cyclooxygenase 2 (COX-2) and histamine were decreased when using this approach, resulting in less post-surgical pain [64].

### Histochemistry of laser-treated gingiva

Kesler et al. [25] conducted an experiment to determine the effects of an Er: YAG laser treatment on the gingival collagen structure and found that this type of laser has the inherent ability to cause tissue shrinkage and enhance collagen remodeling. An immediate treatment did not cause any collagen shrinkage. Their histological findings indicated that the epithelium is removed from the gingiva in most cases with between one to four passes, at 7.0 J/cm2, leaving no scars on the gingival tissues and thus thereby facilitating action in a short time.

Using a diode laser, Yousuf et al. [26] reported that before the laser treatment, the pigmentation had melanin granules in the basal cell layer. Immediately after irradiation, neither inflammatory cells nor any tissue damage, such as carbonization, were observed. After three weeks, a continuous healing process with the proliferation of squamous epithelial cells was noted. The diode laser is a useful and a safe means of treatment, and this type is the preferred type of laser when no other short-pulse lasers are available [28, 10].

Firat et al. [44] studied the histological aspects of wound healing in the gingiva of rats after a low-level laser treatment and found that after seven days of treatment, the laser group had more epithelium as compared to the control group and that over time, blood capillaries, fibroblasts and collagen formation became evident in the laser group. Given that fibroblasts play a major role in the healing of tissue trauma, their increased presence after a laser treatment provided evidence of faster healing with a laser [45, 46].

### Disadvantages of lasers

However, laser surgery does have a number disadvantages. Delayed inflammatory reactions can occur with mild post-operative discomfort lasting for 1-2 weeks. Epithelial regeneration may differ/lag in comparison to that after conventional surgery. In addition, the use of sophisticated equipment makes the treatment quite expensive. A loss of tactile sensitivity can also occur when using lasers [9]. Currently, research is ongoing to make lasers a more clinician-friendly approach.

### Re-pigmentation

The mechanism of re-pigmentation as of now is beyond our understanding, but active melanocytes from the surrounding pigmented tissues are known to migrate to previously treated areas, leading to re-pigmentation [22]. Melanocytes have a reproductive self-maintaining system of cells. When locally depleted, they repopulate, and keratinocyte-derived growth factors (fibroblast growth factor-β) serve as a mitogen. These cells lack desmosomes and possess long dendritic processes that extend between keratinocytes. Melanin is synthesized in melanocytes in small structures known as melanosomes, which are injected into keratinocytes by dendritic processes. Occasionally, adequate tissue removal may not be possible at the gingival margins or interdental regions due to closely placed adjacent teeth which may be affected by the laser. Accordingly, the use of a laser can cause incomplete vaporization of the pigment in such delicate areas, which tends to promote re-pigmentation [41].

## GUMMY SMILE

Gummy smile is caused mainly by excessive gum tissue covering the teeth, an excess of the maxilla, a short upper lip, or hyperactivity of the upper lip that retracts too much during a full smile. When 3mm or more of the gum is displayed upon smiling, it is perceived as unaesthetic [9]. If the cause is overgrowth of the maxillary bone, surgery is required. However, cases involving overgrowth of the gums over teeth may be treated by gum contouring procedures such as gingivectomy and/or gingivoplasty.

### Treatment with lasers

Lasers are a non-invasive procedure that can be used to treat gummy smile and can at times even be used without a local anesthetic. Narayan et al. [29] reported gummy smile correction using a laser with excellent results and emphasized the preservation of the biological width, especially when crowns/veneers are planned after gingival contouring. Sobouti et al. [31] compared a diode laser with a scalpel technique for gingivectomy and found that the bleeding rate was less with the use of a diode laser. They also indicated that using lasers could reduce suturing needs and the demand for analgesics. Shanker et al. [32] also indicated excellent results with diode lasers for treating cases of gingival overgrowth.

Rossman et al. [33] used a CO_2_ laser on monkey gingival tissue and found that at 0.2 seconds of laser exposure, the tissue formed a crater with a center which showed almost complete epithelial destruction, while at 0.5 seconds of treatment, the changes were more severe. The study found that CO_2_ could completely de-epithelialize the gingiva, leaving the connective tissue basically undisturbed and hence promoting selective treatment with enhanced precision. Marques et al. [34] used a low-power gallium pulsed laser to analyze the protein synthesis of fibroblasts and reported ultrastructural changes in cytoplasmic organelles, especially the mitochondria (edematous) and RER (marked dilation), indicating that this type can change the ultrastructure of cultured fibroblasts.

## PLASMA AS A NOVEL TREATMENT TECHNIQUE

**Figure 6:**
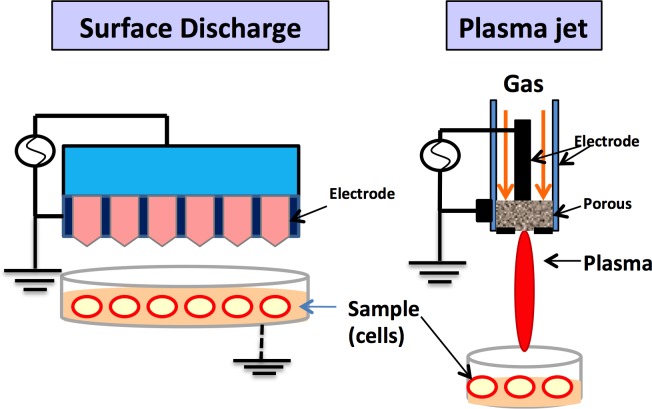
There are various devices for plasma treatment The figure depicts dielectric plasma discharge and plasma jet. They can cause changes in the human fibroblasts and other cells and also aid in wound healing without post-operative pain.

The term plasma, coined by Irving Langmuir, refers to the fourth state of matter (after the solid, liquid and gas forms). Non-thermal plasma (Figure 6) consists of partially ionized gases (helium, argon, oxygen, nitrogen may be used) with free electrons, generated at a low temperature (less than 400C). The use of plasma has recently expanded in the fields of medicine and dentistry [53]. The mechanism of action of plasma is based on release of free radicals and reactive species (e.g., reactive oxygen and nitrogen species, i.e., ROS and RNS) [54]. These radicals control the cell redox signaling pathway and can be used for sterilization and wound healing and apoptosis of cancer cells, but their high concentration can have adverse effects on the cells. It has been found that non-thermal plasma can produce significant amounts of ozone [55, 56]. This generated ozone in aqueous media further generates biologically active ROS and RNS.

### Plasma in medicine and dentistry

Non-thermal plasma has been studied and used in the medical field, mainly for the treatment of cancer. Previously, plasma was used in medicine in relation to heat and thermal treatments for sterilization and to control bleeding. Electrocautery is one of the uses of thermal plasma, but it can result in the charring of tissue [58]. Non-thermal plasma is a novel technique with selective action no damage to the surrounding tissue. Skin wounds and superficial skin infections have been treated successfully with plasma jets. At low temperatures, plasma has a positive effect on wound healing. Nosenko et al. [60] demonstrated the proliferation of fibroblasts upon exposure of 5 min using a plasma device.

In dentistry, plasma devices have been researched recently with promising results. A plasma-thorn device was fabricated and successfully used to inactivate *B. aureus* on Lactobacillus agar [61]. This in vitro result can be utilized for root canal treatment therapy. Lu et al. [62] tested a plasma jet for disinfection after a root canal. Plasma has also been used to increase the bonding at the enamel-dentin interface and to remove smear layers. The use of plasma as a treatment of diseased periodontal tissues was also reported recently. Periodontal pockets [70] can be treated with plasma [71] in a manner similar to how lasers are currently used [64]. Sun et al. [72] examined the bactericidal effects of plasma to examine their potential to disinfect gingival crevices. Target treatments can be provided for diseased soft tissues containing bacteria, viruses and fungi without causing damage to the surrounding healthy tissue. Studies are also underway to investigate cell proliferation, attachment and osteogenesis following a treatment with non-thermal plasma. Wound healing and the angiogenesis effects of non-thermal plasma have been widely studied in the medical field. Using the same principles, wound healing with respect to dental hyperpigmentation and gummy smile treatment represents another potential area of application. Stoffels et al. [69] studied the successful proliferation of plasma-induced fibroblasts. Friedman et al. [58] studied the positive NO gas effects on keratinocytes, collagen synthesis and phagocytosis.

### Wound healing after laser and plasma treatment

Wound healing takes places in three different phases: the substrate phase, the proliferative phase and the remodeling phase. The maximum effect of the laser occurs in the proliferative phase [51], which consists of angiogenesis, granulation tissue formation, and epithelialization. A laser treatment results in wound healing with minimal scar formation.

**Figure 7:**
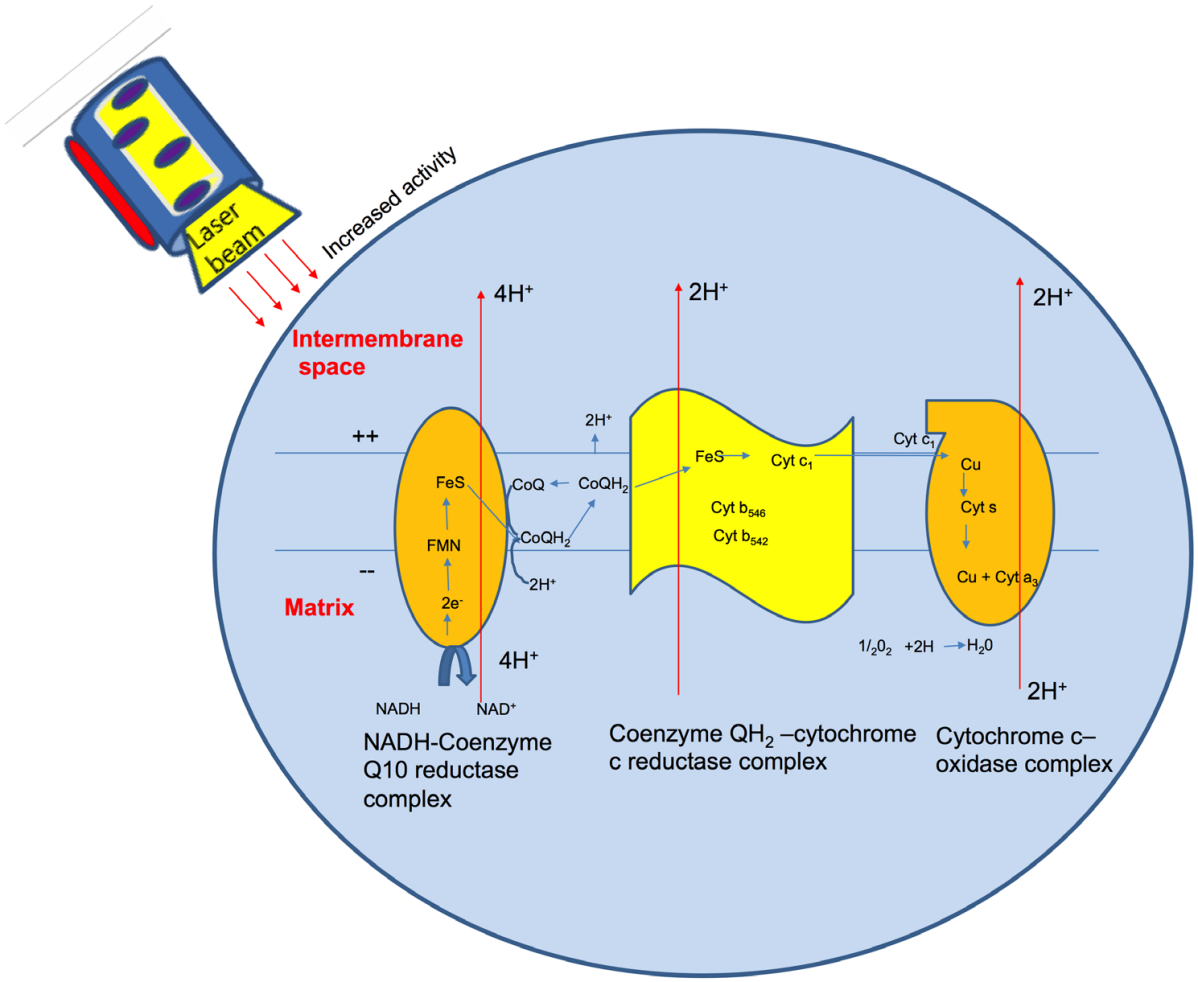
Electron activity within the cytochrome oxidase molecule The cytochrome oxidase is a photoreceptor of light. Its internal activity is increased with laser treatment.

Photo-bio modulation is known to occur due to changes in the mitochondrial activity within cells as an after-effect of a laser or plasma treatment (as plasma also generates various ROS and RNS species). Cytochrome oxidase has been studied as a photoreceptor of light. Irradiation with a laser can activate the redox metal centers of cytochrome oxidase, showing an increased level of electron transfer activity (Figure 7) and in turn causing increased mitochondrial respiration [51].

**Figure 8:**
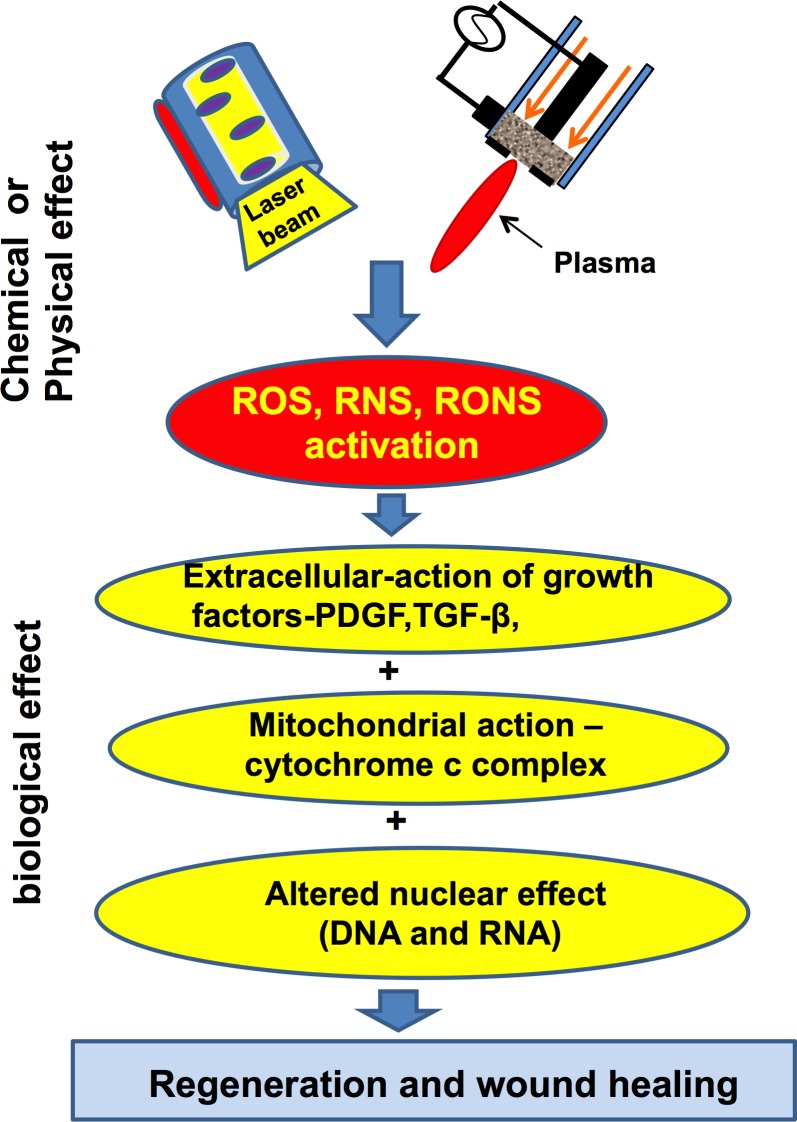
Laser and Non-thermal plasma can activate the free radicals (RNS, ROS), which lead to a cascade of events resulting in faster wound healing and regeneration of cells and tissues

With an effective laser treatment, fibroblasts and collagen increase, resulting in more rapid wound closure. Hunt et al. [52] suggested a potential mechanism by which LLLT affects the regeneration and oral wound healing processes. Laser and non-thermal plasma treatments lead to the activation of free radicals (e.g., RNS, ROS), resulting in the activation of the TGF-β gene and growth factors [54] and altering the cell at the DNA/RNA level (Figure 8). In this way, the re-epithelialization of human gingival or skin tissue takes place with quicker healing without any side effects, pain or discomfort to patients. Till now there is lack of availability of comparison studies between laser and non- thermal plasma therapy for wound healing process. However, various studies indicate that the method of wound healing for both the modalities is somewhat similar [55]. As a result of plasma treatment, there is alteration in the cell/plasma membrane with induction of intracellular reactive oxygen/nitrogen radicals. Low dose of plasma is effective for proliferation, migration and repair of damaged cells/DNA, but at high doses can cause cell cycle arrest or apoptosis. Plasma can also have positive effect on the angiogenesis and cell proliferation. Haertel et al. [56] reported formation of new micro vessels after plasma treatment. The effect can lead to modification of cell adhesion molecules which in turn affects the cell matrix interaction, and hence initiate faster wound healing.

## CONCLUSIONS

Laser treatments for gingival pigmentation and gummy smile are safe and comfortable for patients. Dental lasers enhance and improve the classical procedures done with burs or scalpels, and they offer a much wider range of treatment protocols with greater precision of control. Laser treatments are also friendlier to patients and dentists. Therefore, it is expected that their use will increase with time.

The single-visit laser treatment has become the need of the hour, referring to a rapid treatment associated with ease of use and complete patient satisfaction. The diode laser (810-1064 nm) has become popular in dentistry due to its small size, lower cost than other lasers, fiber optic delivery method and ease of use for surgery on oral soft tissue.

Plasma is being used increasingly in medicine and lately has become popular in dentistry as well. Plasma device sizes on the micrometer scale opens doors to many potential applications, such as the treatment of multiple internal cavities or complex hollow structures. This type of treatment can inhibit bacterial growth significantly in the root canal, resulting in maximum sterilization during dental treatments [61]. Odontophobia, i.e., dental fear, is the main reason behind children and some adults avoiding a visit to their dentist. The use of plasma-aided devices will help to alleviate this fear, as they provide painless treatments and help to secure proper dental care to all [63].

Plasma can be used effectively in wet and dry environments and in the presence of blood, gingival crevicular fluid and saliva [64]. Plasma-aided dental devices may replace exciting technologies and emerge as a future non-surgical, non-invasive treatment modality, especially in periodontal dentistry. They can regenerate and differentiate periodontal stem cells and thus show potential as a future dental therapy, and they facilitate successful gingival treatments such as that for gummy smile, resulting in the more rapid generation of various dental-related cells (e.g., fibroblasts, collagen) with the least amount of post-operative pain to the patient [65].

Plasma is available in various types like dielectric barrier discharge, plasma jet, plasma torch & barrier coronal discharge. Research is being done in the research centers and medical industry with advent of new devices for fast and easy treatment. In medical field, argon plasma torch (MicroPlaSter) has been introduced for a randomized controlled trial for patients with chronic infected wounds with well tolerated healing results. Another plasma device,’plasmafone’ has been tested for treatment of diabetic foot in human patients with successful results [66]. Plasma ONE, a medical device is commercially available for surgical use for dermatology, urology and gynecology patients. It is also being used in dentistry for sterilization and biofilm removal from the tooth surface. Research has shown positive plasma effects on root sterilization, disinfection and tooth whitening using plasma jets and plasma torches [67]. The kINPen argon plasma jet is available as a commercial medical device, with strong antimicrobial activity and (skin) wound healing properties [68]. Recent study on jet (Plasque) developed by Plasma Bioscience Research Center of Korea showed many dental applications and is under process for clinical trials.

The costs associated with the use of plasma devices are much lower than those of laser devices, making it plasma devices much more suitable for surgical purposes. In comparison to a laser treatment, where delayed wound healing and rare post-treatment discomfort can arise, the use of plasma has shown no such deleterious effects during or after treatment. Research is being done to include more plasma devices for dental treatment and sterilization procedures.

Laser and plasma torches [73] are already being used for welding in the manufacturing industry. Currently, ongoing research focuses on utilizing laser and plasma technologies in combination for medical microsurgery and other procedures. Laser induced plasma channel utilizes a laser with plasma whereby a short laser pulse causes release of laser in the air which ionizes the surrounding gases. This results in the formation of a plasma (formation of plasma channel) and it causes ablation of the target surface when focused on it [74]. It is possible that in the future, such technology will be available for dental soft tissue procedures as well (hyperpigmentation and gummy smile), at a price affordable for commercial use.
